# Prolonged nightly fasting and lower-extremity functioning in community-dwelling older adults

**DOI:** 10.1017/S0007114520005218

**Published:** 2021-11-14

**Authors:** Daniela B. Estrada-deLeón, Ellen A. Struijk, Francisco Félix Caballero, Mercedes Sotos Prieto, Fernando Rodríguez-Artalejo, Esther Lopez-Garcia

**Affiliations:** 1 Department of Preventive Medicine and Public Health. School of Medicine, Universidad Autónoma de Madrid, Madrid, Spain; 2 IdiPaz (Instituto de Investigación Sanitaria Hospital Universitario La Paz), Madrid, Spain; 3 CIBERESP (CIBER of Epidemiology and Public Health), Madrid, Spain; 4 Department of Environmental Health, Harvard T.H. Chan School of Public Health, Boston, MA, USA; 5 IMDEA-Food Institute, CEI UAM+CSIC, Madrid, Spain

**Keywords:** Time-restricted feeding, Intermittent fasting, Physical function, Short Physical Performance Battery, Older adults, Cross-sectional studies

## Abstract

It is unknown if time-restricted feeding confers a protective effect on the physical function of older adults. The aim of this study was to assess prolonged nightly fasting in association with performance-based lower-extremity function (LEF) in a large population of community-dwelling older adults. A cross-sectional study was carried out among 1226 individuals ≥64 years from the Seniors-ENRICA-II (Study on Nutrition and Cardiovascular Risk in Spain) cohort. Habitual diet was assessed through a validated diet history. Fasting time was classified into the following categories: ≤9, 10–11 and ≥12 h/d (prolonged nightly fasting). Performance-based LEF was assessed with the Short Physical Performance Battery (SPPB). After adjusting for potential confounders, a longer fasting period was associated with a higher likelihood of impaired LEF (OR for the second and third categories *v.* ≤ 9 h/d fasting: 2·27 (95 % CI 1·56, 3·33) and 2·70 (95 % CI 1·80, 4·04), respectively; *P*_trend_ < 0·001). Fasting time showed a significant association with the SPPB subtests balance impairment (OR for highest *v.* shortest fasting time: 2.48; 95 % CI 1·51, 4·08; *P*_trend_ = 0·001) and difficulty to rise from a chair (OR 1·47; 95 % CI 1·05, 2·06; *P*_trend_ = 0·01). The risk associated with ≥12 h fasting among those with the lowest levels of physical activity was three times higher than among those with ≤9 h fasting with the same low level of physical activity. Prolonged nightly fasting was associated with a higher likelihood of impaired LEF, balance impairment, and difficulty to rise from a chair in older adults, especially among those with low levels of physical activity.

Several types of intermittent fasting have emerged as an alternative to energetic restriction and to a prolonged period of fasting because this diet strategy is relatively easy to maintain^(1)^ and there is some evidence from studies in animals and humans that it might improve overall health^(2)^ and body composition^(3)^, protects against cardiometabolic risk factors^(4,5)^ and extends life span^(6)^. A specific type of intermittent fasting is time-restricted feeding, in which the daily eating period is limited to 12 h or less per d. Studies in humans have shown that time-restricted feeding is associated with weight loss^(7)^, fat mass decrease^(8)^, metabolic disease risk reduction^(4)^ and improvement in glycaemic response^(9)^.

Several physiological mechanisms might explain how intermittent fasting can affect metabolic pathways^(10)^. Generally, after 12 h of food deprivation, the hepatocytes glycogen stores are depleted and the liver shifts from glucose storage to gluconeogenesis and produces ketone bodies from fatty acids, such as *β*-hydroxybutyrate and acetoacetate, to satisfy the energy requirements of brain cells and to preserve muscle mass and muscle function^(2,11,12)^. Thus, individuals engaging in intermittent fasting might maintain or even enhance their physical function^(13–15)^. Although the relatively short duration of fasting in time-restricted fasting may not lead to ketosis, it can trigger other processes such as autophagy, where damaged macromolecules and organelles can be cleared to preserve normal cell function^(16)^. Moreover, intermittent fasting may have an impact on the circadian rhythm that regulates metabolism, energetics and sleep–wake cycles^(17,18)^.

Yet, evidence about the effects of intermittent fasting in free-living populations is scarce. Specifically, the impact of time-restricted fasting on physical function in older adults is unknown. In this population, time-restricted fasting happens unintentionally as prolonged nightly fasting and may pose individuals at higher risk of malnutrition. In spite of the fact that the need of energy intake is substantially reduced with age, macronutrient needs in older adults are similar or even greater than in younger adults in order to prevent sarcopenia and frailty^(19)^. We hypothesised that prolonged nightly fasting could be related to functional impairment in older adults. Therefore, the aim of this study was to assess prolonged nightly fasting in association with performance-based lower-extremity function in a large population of community-dwelling older adults.

## Materials and methods

### Study design and participants

We performed a cross-sectional analysis of data obtained from the Seniors-ENRICA-II (Study on Nutrition and Cardiovascular Risk in Spain) cohort, among 3273 community-dwelling individuals aged 64 years or older residing in the city of Madrid and four surrounding towns: Alcalá de Henares, Alcorcón, Getafe and Torrejón de Ardoz. This cohort followed the same design as the Seniors-ENRICA-I^(20)^. The study participants were selected by random sampling sex- and district-stratified among all individuals with a national healthcare card between 2015 and 2017. Given that all people residing in Spain are entitled to free healthcare, the list of card holders closely approximates the entire resident population of Madrid. Information was collected by trained staff in three stages: (1) a computer-assisted telephone interview on socio-demographic data, lifestyle, health status and morbidity; (2) a first home visit to perform a physical examination and obtain biological samples and (3) a second home visit, conducted 7 d after the first one, to take a diet history and obtain other questionnaire data. Participants were suggested to have a proxy to accompany them for the home visits to help them respond to the diet history and to help them feel comfortable and safe. At the end of the first home visit, an accelerometer was located on the wrist of participants and was returned during the second visit, 7 d apart^(21)^. Study participants gave written informed consent. The study was approved by the Clinical Research Ethics Committee of ‘La Paz’ University Hospital in Madrid (Spain).

### Study variables

#### Diet

Trained interviewers obtained information on food consumption through a validated computerised dietary history, which was developed from the one used in the EPIC-Spain cohort study^(22)^. The diet history included 880 foods along with 127 sets of photographs to help estimate the serving size. This instrument collects food consumption by occasions of intake and accommodates habitual diet information to a 24-h format, by asking participants to indicate the time in which they usually had their meals, including snacks. Fasting time was defined as the window (in h) between the last food ingested before going to sleep and the first food consumed upon getting up in the morning. Participants were classified in three categories: those reporting a fasting time of ≤9 h/d, those with fasting time between 10 and 11 h/d and those with fasting time ≥12 h/d, which can be considered prolonged nightly fasting^(12)^.

Nutrient intakes were estimated using standard food composition tables for the Spanish population^(23)^. The Mediterranean Diet Adherence Screener score was also estimated for all participants in order to define the overall diet quality^(24)^. This score ranged from 0 to 14, and higher scores indicated greater adherence and thus higher diet quality. The validity of the diet history was evaluated by comparing results from this instrument with seven 24-h recalls over a 1-year period among a subsample of participants; the observed correlations ranged between 0·27 and 0·71 across food groups and nutrients, which are in line with those for most instruments assessing self-reported diet in population studies^(25)^.

#### Physical function

We assessed lower-extremity function with the Short Physical Performance Battery (SPPB), which includes three timed tasks: gait speed, standing balance and ability to rise from a chair^(26,27)^. Gait speed was calculated as the time that a participant walked at usual pace across 2·44 m, timed from a standing position. The standing balance test evaluated the time that participants could maintain three hierarchical tandem positions: side-by-side, semitandem and tandem positions. The ability to rise from a chair was assessed by the time required to stand up and sit down from a chair five times consecutively as fast as possible and with their arms folded across their chest. Each test was scored from 0 to 4, and the total SPPB score was the sum of these three components (range 0–12); a higher score indicates better physical performance. We used the standard cut-off of ≤9 to define impaired lower-extremity function (ILEF). In addition, balance impairment, difficulty to raise from a chair and slow gait were defined as a score of ≤3 in each scale^(26)^.

#### Other variables

We obtained information on age, sex, education (primary, secondary or university level), smoking status (never, former or current smoker), sedentary behaviour (h/week spent watching television), alcohol intake (g/d), sleep duration (h/d) and energy intake (kJ/d). Physical activity (metabolic equivalent-h/week) was assessed with the ActiGraph GT9X (ActiGraphInc) accelerometer, which was asked to be worn for seven consecutive days^(21)^. Weight and height were measured under standardised conditions, using electronic scales and portable extendable stadiometers. BMI was calculated as weight (kg) divided by height squared (m^2^) and classified as <25, 25–29·9 or ≥30 kg/m^2^. In addition, the following physician-diagnosed diseases were self-reported: musculoskeletal disease, CVD, diabetes, cancer, chronic lung disease and depression requiring treatment.

### Statistical analyses

From the 3273 participants in the cohort, we selected the 1732 participants with information on timing of food consumption. Then, we excluded one participant with implausibly energy intake and another one without information on educational level. Additionally, we excluded 454 individuals without measurement of SPPB and fifty without accelerometry. Thus, the analyses were conducted with 1226 individuals. There is no available power calculation for these analyses since data were obtained from a population study with multiple *a priori* hypothesis based on lifestyles and healthy ageing; the analyses of this manuscript were performed only among the available sample with information on habitual fasting.

Participants’ characteristics were summarised as means and standard deviations for continuous variables and as percentages for categorical variables. Differences in characteristics across participants in the different fasting categories were tested by ANOVA for quantitative variables and by the *χ* ^2^ test for categorical variables. Logistic regression was used to estimate the OR and the 95 % CI for the association between different fasting categories and ILEF.

Several logistic models were built with sequential adjustment for potential confounders: the first one included sex, age and energy intake; the second one was additionally adjusted for educational level, smoking status, sedentary behaviour, alcohol intake (g/d), BMI, morbidity, sleep duration, protein intake and Mediterranean Diet Adherence Screener score and the third model was also adjusted for physical activity since this variable could also be considered a potential mediator in the studied association. Additionally, we assessed the linear dose–response relationship (*P*_trend_) by modelling fasting time as a continuous variable. To test non-linear risk trends, we used three knot-restricted cubic splines for fasting time and the risk of ILEF. Test for non-linearity used the likelihood ratio test, comparing the model with only the linear term to the model with the linear and the cubic spline terms. The association between prolonged nightly fasting and each SPPB component was also evaluated.

We replicated the analyses for the associations by strata of sex, BMI (<30, ≥30 kg/m^2^), sleep time (above and below the median), depression (no, yes), energy intake (above and below the median), total protein intake (above and below the median) and diet quality (above and below the median) to assess the robustness of the results. The possible modifying effect of these variables on the studied association was assessed with the likelihood-ratio test. Finally, we examined the joint effect of fasting categories and physical activity on ILEF, by considering physical activity in tertiles and using as reference category the simultaneous condition of a fasting time of ≤9 h/d and being in the highest tertile of physical activity. Last, in a sensitivity analysis, we examined the impact of depression in the studied association by excluding participant with a diagnosis of this disease. Statistical significance was set at two-tailed *P* < 0·05. Analyses were conducted using Stata (version 15.1; Stata Corp.)

## Results

Characteristics of study participants according to the three fasting time categories are presented in Table 1. Those with prolonged nightly fasting (≥12 h) were significantly older, more often women, never smokers, more likely to have a diagnosis of depression and reported more hours of sleep, compared with those in the shortest fasting time category. In addition, total energy, protein and fat intake were lower in those with prolonged nightly fasting, although adherence to the diet quality index was similar across groups.

**Table 1.** Baseline characteristics of the study participants (*n* 1226) by categories of fasting time (Mean values and standard deviations; numbers and percentages)

	≤9 h fasting		10 h–11 h fasting		≥12 h fasting		*P* *
	Mean	sd	Mean	sd	Mean	sd	
*n*	578		388		260		
Age (years)	70·8	3·9	70·6	3·7	71·7	4·2	0·002
Sex							
Men (%)	54		53		42		0·005
Educational level (%)							
≤Primary	58		44		52		0·001
Secondary	17		22		20		
University	25		34		28		
Smoking status (%)							
Current smoker	11		9		5		0·02
Former smoker	42		45		38		
Never smoker	47		46		57		
Television watching (h/week)	22·1	10·3	20·9	9·9	21·1	10·0	0·16
Physical activity							
MET-h/week	67·4	34·4	66·7	36·7	63·7	35·8	0·37
BMI (kg/m^2^, %)							
<25	30·3		29·4		29·6		0·17
25–29·9	42·6		49·0		47·7		
≥30	27·1		21·6		22·7		
Diagnosed morbidity (%)							
Musculoskeletal disease†	45		40		43		0·33
CVD‡	3		5		5		0·23
Diabetes	25		25		23		0·84
Cancer	3		2		4		0·18
Chronic lung disease	7		8		8		0·87
Depression	6		6		13		0·001
Sleep duration (h/d)	6·8	1·3	6·8	1·2	7·1	1·4	0·03
Energy (kJ/d)	8075	1615	7707	1155	7648	1259	<0·001
Protein (g/d)	93·7	18·3	90·3	16·2	88·7	15·3	<0·001
Protein (g/kg per d)	1·3	0·3	1·2	0·2	1·3	0·3	0·05
Fat (g/d)	73·3	23·6	69·6	18·9	68·7	21·2	0·005
Carbohydrate (g/d)	205·6	42·2	194·4	36·0	197·7	33·2	<0·001
Alcohol intake (g/d)	10·0	14·2	10·5	13·2	8·5	11·6	0·18
MEDAS score	6·6	1·6	6·5	1·6	6·5	1·7	0·63

MEDAS, Mediterranean Diet Adherence Screener.

* ANOVA test was used for quantitative variables and the *χ* ^2^ test for categorical variables.

† Osteo-arthritis, arthritis and hip fracture.

‡ IHD, stroke and heart failure.

In Table 2, a significant association between a longer fasting time and ILEF was found; in age-, sex- and energy intake-adjusted models, the OR for 10–11 and ≥12 h/d of fasting were 2·06 (95 % CI 1·44, 2·94) and 2·56 (95 % CI 1·76, 3·73), respectively, using ≤9 h as the reference category (*P*_trend_ < 0·001). After additional adjustment for other potential confounders including physical activity, we obtained similar results (OR 2·27 (95 % CI 1·56, 3·33); 2·70 (95 % CI 1·80, 4·04); *P*_trend_ < 0·001). Fasting time also showed a significant association with balance impairment (OR for longest *v*. shortest fasting time: 2·48; 95 % CI 1·51, 4·08; *P*_trend_ = 0·001) and difficulty to rise from a chair (OR 1·47; 95 % CI 1·05, 2·06; *P*_trend_ = 0·01). Slow gait speed was not associated with fasting.

**Table 2.** Association between fasting time categories and impaired lower-extremity function (ILEF), balance impairment, difficulty to rise from a chair and slow gait (Odds ratios and 95 % confidence intervals, *n* 1226)

	≤9 h fasting	10–11 h fasting		≥12 h fasting		*P*_trend_
		OR	95 % CI	OR	95 % CI	
*n*	578	388		260		
ILEF, *n* cases	74	84		76		
Model 1*	1·00	2·06	1·44, 2·94	2·56	1·76, 3·73	<0·001
Model 2†	1·00	2·25	1·55, 3·27	2·77	1·86, 4·12	<0·001
Model 3‡	1·00	2·27	1·56, 3·33	2·70	1·80, 4·04	<0·001
Balance impairment, *n* cases	41	30		43		
Model 1*	1·00	1·21	0·73, 1·99	2·39	1·49, 3·84	<0·001
Model 2†	1·00	1·20	0·71, 2·02	2·60	1·58, 4·26	<0·001
Model 3‡	1·00	1·18	0·69, 1·99	2·48	1·51, 4·08	0·001
Difficulty to raise from a chair, *n* cases	365	261		183		
Model 1*	1·00	1·24	0·94, 1·63	1·38	1·00, 1·90	0·03
Model 2†	1·00	1·34	1·01, 1·79	1·47	1·06, 2·06	0·01
Model 3‡	1·00	1·37	1·03, 1·82	1·47	1·05, 2·06	0·01
Slow gait, *n* cases	148	95		87		
Model 1*	1·00	0·93	0·69, 1·26	1·26	0·91, 1·74	0·26
Model 2†	1·00	0·91	0·66, 1·24	1·28	0·91, 1·79	0·25
Model 3‡	1·00	0·91	0·67, 1·24	1·27	0·90, 1·78	0·27

* Model 1: OR (95 % CI) adjusted for sex, age and energy intake (quintiles of kJ/d).

† Model 2: OR (95 % CI) additionally adjusted for educational level (≤primary, secondary or university), smoking status (never, former, current smoker), sedentary behaviour (tertiles of h/week watching television), alcohol intake (quintiles of g/d), BMI (<25, 25–29·9, ≥30 kg/m^2^), morbidity (musculoskeletal disease, CVD, cancer, diabetes, chronic lung disease and depression), sleep duration (tertiles of h/d), protein intake (quintiles of g/d) and for Mediterranean Diet Adherence Screener score (tertiles).

‡ Model 3: OR (95 % CI) additionally adjusted for physical activity (tertiles of MET-h/week).

**Fig. 1.**
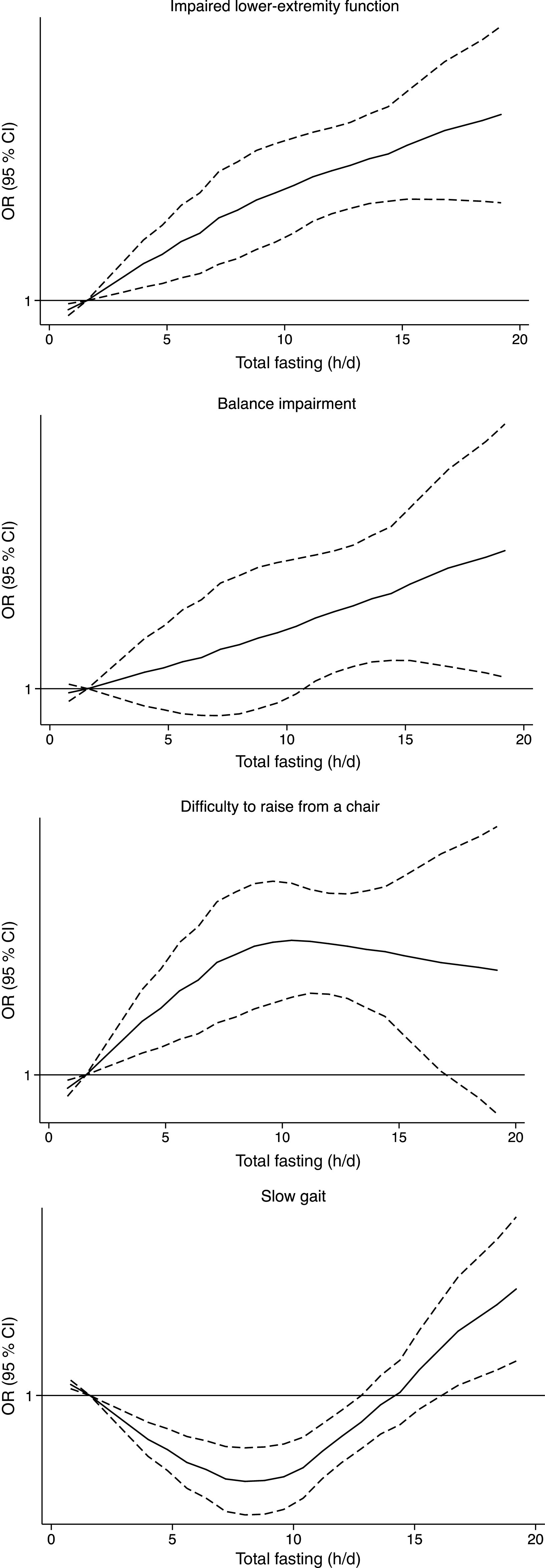
Multivariable-adjusted spline curves for the relation between fasting time and the risk of impaired lower-extremity function. Adjusted for sex, age, educational level (≤primary, secondary or university), smoking status (never, former, current smoker), sedentary behaviour (h/week watching television), alcohol intake (quintiles of g/d), BMI (<25, 25–29·9, ≥30 kg/m^2^), morbidity (musculoskeletal disease, CVD, cancer, diabetes, chronic lung disease and depression), sleep duration (tertiles of h/d), energy intake (quintiles of kJ/d), protein intake (quintiles of g/d), Mediterranean Diet Adherence Screener score (tertiles), and physical activity (tertiles of MET-h/week).

**Fig. 2.**
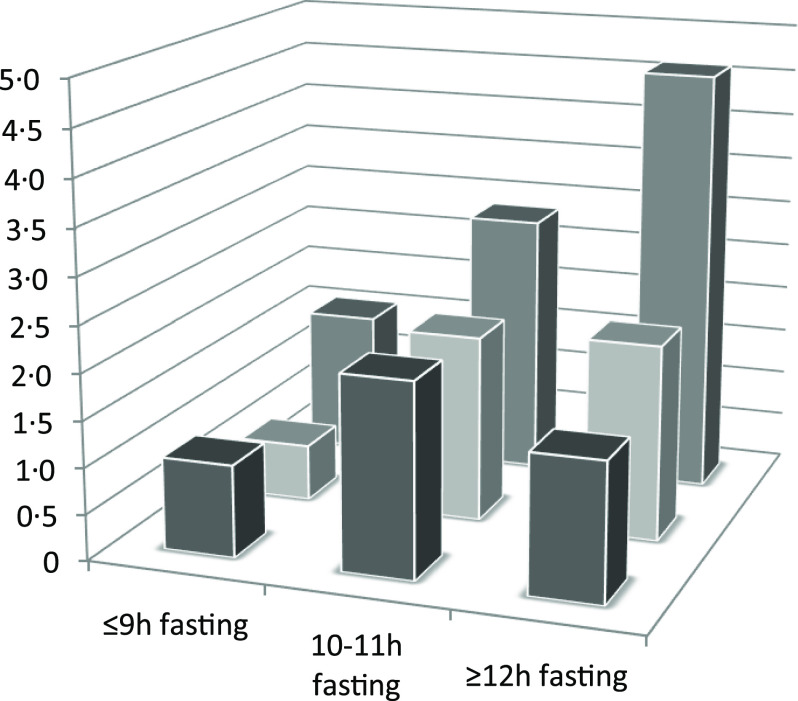
Odds ratios for the joint association of fasting time and physical activity categories with impaired lower-extremity function. Adjusted for sex, age, educational level (≤primary, secondary or university), smoking status (never, former, current smoker), sedentary behaviour (h/week watching television), alcohol intake (quintiles of g/d), BMI (<25, 25–29·9, ≥30 kg/m^2^), morbidity (musculoskeletal disease, CVD, cancer, diabetes, chronic lung disease and depression), sleep duration (tertiles of h/d), energy intake (quintiles of kJ/d), protein intake (quintiles of g/d) and Mediterranean Diet Adherence Screener score (tertiles). Cut-off points to define levels of physical activity were: ≤57·5 (low), 57·6–82·0 (intermediate) and ≥82·1 MET-h/week (high). Reference category included participants with ≤9 h/d of fasting time and with high physical activity level. 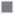, High physical activity; 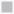, intermediate physical activity; 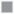, low physical activity.

The dose–response association between the fasting time and ILEF is assessed in Fig. 1; the test for non-linearity was not significant, except for gait speed (*P* ≤ 0·001). The association between different fasting categories and ILEF was assessed by strata of sex, BMI, sleep, depression, energy intake, total protein intake and diet quality and is shown in Table 3. No significant differences were identified across the strata (*P*_interaction_ > 0·05 in all cases). In addition, when fasting time and physical activity were considered simultaneously, ≥12 h fasting had double risk of ILEF than those with <9 h of fasting (OR 1·50, 95 % CI 0·62, 3·60 *v.* OR 1) among those with high levels of physical activity. The risk associated with ≥12 h fasting among those with the lowest levels of physical activity was three times higher than among those with ≤9 h fasting with the same low level of physical activity (OR 4·60, 95 % CI 1·42, 14·84 *v.* OR 1·55, 95 % CI 0·49, 4·88) (*P*_interaction_ < 0·001) (Fig. 2). Last, sensitivity analyses by excluding participants with depression yielded similar associations than in the main tables (online Supplementary Table S1).

**Table 3.** Association between fasting time categories and impaired lower-extremity function, by specific subgroups of older adults* (Odds ratios and 95 % confidence intervals, *n* 1226)

	≤9 h fasting	10–11 h fasting		≥12 h fasting		*P*_trend_	*P*_interaction_
		OR	95 % CI	OR	95 % CI		
*n*	578	388		260			
Sex							
Men (*n* 627)	1·00	1·41	0·77, 2·59	2·63	1·34, 5·17	0·006	0·24
Women (*n* 599)	1·00	3·06	1·75, 5·34	3·33	1·86, 5·97	<0·001	
BMI							
<30 kg/m^2^ (*n* 926)	1·00	2·14	1·32, 3·46	2·48	1·47, 4·18	<0·001	0·70
≥30 kg/m^2^ (*n* 300)	1·00	1·97	0·95, 4·08	2·82	1·30, 6·10	0·006	
Sleep duration							
<Median (*n* 613)†	1·00	1·87	0·96, 3·63	3·13	1·51, 6·47	0·002	0·89
≥Median (*n* 613)†	1·00	2·36	1·43, 3·91	2·67	1·58, 4·51	<0·001	
Depression							
No (*n* 1130)	1·00	2·24	1·50, 3·34	2·92	1·88, 4·53	<0·001	0·94
Yes (*n* 96)	1·00	2·35	0·28, 20·0	4·28	0·54, 34·1	0·17	
Energy intake							
<Median (*n* 613)†	1·00	3·29	1·85, 5·85	3·28	1·79, 6·00	<0·001	0·10
≥Median (*n* 613)†	1·00	1·31	0·74, 2·32	2·46	1·32, 4·59	0·006	
Protein intake							
<Median (*n* 613)†	1·00	2·65	1·51, 4·64	2·50	1·38, 4·50	0·002	0·34
≥Median (*n* 613)†	1·00	1·65	0·91, 2·99	2·96	1·57, 5·57	0·001	
MEDAS score							
<Median(*n* 590)†	1·00	2·84	1·62, 5·00	3·58	1·93, 6·64	<0·001	0·28
≥Median (*n* 636)†	1·00	1·67	0·94, 2·96	2·08	1·14, 3·80	0·014	

MEDAS, Mediterranean Diet Adherence Screener.

* From logistic regression models adjusted for sex, age, educational level (≤primary, secondary or university), smoking status (never, former, current smoker), sedentary behaviour (h/week watching television), alcohol intake (quintiles of g/d), BMI (<25, 25–29·9, ≥30 kg/m^2^), morbidity (musculoskeletal disease, CVD, cancer, diabetes, chronic lung disease and depression), sleep duration (tertiles of h/d), energy intake (quintiles of kJ/d), protein intake (quintiles of g/d), MEDAS score (tertiles) and physical activity (tertiles of MET-h/week) except for the stratification variable.

† Median sleep duration: 7 h; median energy intake: 7669 kJ; median protein intake: 90·3 g; median MEDAS score: 7.

## Discussion

In this cross-sectional study of community-dwelling older adults, a fasting time of more than 12 h/d was associated with a higher risk of ILEF, balance impairment and difficulty to rise from a chair. These associations were robust across different strata, including sex, BMI, having depression, energy intake, total protein intake and diet quality. In addition, these results held on different levels of physical activity, which suggests that despite the beneficial effect of physical activity on function, fasting time has an independent association with this endpoint in older adults. Notwithstanding this, high physical activity could compensate for part of the excess frequency of ILEF associated with longer fasting.

Most of the evidence on the effects of intermittent fasting has been obtained in animal studies^(4–6)^. Human studies have been restricted to younger adults in experimental designs with extreme intermittent fasting and a small number of participants^(7–9)^. To our knowledge, this is the first study assessing fasting in association with ILEF. Our findings of an increased risk of balance impairment in participants on prolonged nightly fasting are in line with previous publications in older adults who practiced Ramadan fasting^(28,29)^. A study among fifteen older men showed that fasting approximately 17 h/d for 4 weeks had a detrimental effect on postural balance performance and in simple reaction time^(28)^. Another study compared the effects of Ramadan fasting on postural control between a group of twelve participants with a history of at least two spontaneous and unexpected falls during the previous year and a group of twelve non-fallers. Ramadan fasting had similar adverse effects on postural control in both groups that could lead to balance dysfunction and mobility limitation in older adults^(29)^. Despite Ramadan fasting is a common variety of time-restricted fasting, it is of note that fasting time is approximately 12 h/d from dawn to sunset^(17)^, which contrasts with prolonged nightly fasting in our study and with usual time-restricted diets.

In our population, fasting time was not associated with the risk of slow gait. The tests included in the SPPB reflect different abilities. Balance and difficulty to raise from a chair reflect diminished muscle mass and energy deficit, and slow walking speed integrates these aspects with neurological control^(26)^. We can hypothesise that prolonged nightly fasting may have a detrimental effect on some aspects related to physical function, such as muscle mass and strength but not in others related to neurological function. Of note, in a small non-randomised clinical trial among ten overweight older adults who were asked to fast for 16 h/d during 4 weeks, slow gait, grip strength and quality of life were improved, although this may be driven by the weight loss that all participants experienced, which was the main target of the intervention^(13)^. This study lacked of a control group, so causal effects were not demonstrated. In another recent clinical trial^(30)^, a maintained 14 h/d of fasting for 6 weeks without weight loss did not influence lean mass, bone density or nutrient intake. In our results, stratification for BMI level showed a similar association of prolonged nightly fasting with ILEF; however, since this was a cross-sectional study, we could not assess whether weight loss mediated the study association.

It is well known that the best approach to counteract physical function impairment is regular practice of physical activity^(31–33)^. In our study, although participants with a higher level of physical activity had a lower risk of ILEF than their less active counterparts, the augmented risk of ILEF associated with longer fasting was present at all physical activity levels. Adequate dietary protein intake is also strongly related to physical function. Proteins have been associated with maintenance of physical function, muscle mass and bone mass density^(34–38)^ and have been shown to prevent frailty in older adults^(39)^. A previous study found that a higher intake of dietary animal protein in combination with physical activity was associated with higher skeletal muscle mass and a lower likelihood of functional limitation development^(40)^. In our study, participants on prolonged nightly fasting had lower intake of dietary protein and energy in comparison with participants with fasting time <9 h/d; however, all participants in the three fasting groups had a protein intake above the current RDA, of 0·8 g protein/kg per d^(41,42)^. Despite participants on prolonged nightly fasting were meeting their adequate intake of proteins, prolonged fasting might reduce the intake of other relevant nutrients for older adults resulting in reduced function.

The involuntary loss of appetite that leads to reduced food intake is a common problem in older adults, usually known as anorexia of ageing^(43)^. Several factors for anorexia of ageing have been described^(44)^, including hearing and vision impairment, specific medical conditions such as gastrointestinal diseases, poly-medication, social factors including social isolation and depression. In our study, participants on prolonged fasting presented more depression than those who did not fast ≥12 h. A recent study in community-dwelling older adults found a higher prevalence of depression in participants with poor appetite, in comparison with those with normal appetite. Also, they found an association between poor appetite and lower skeletal muscle mass, lower grip strength and low muscle mass^(45)^. However, in our sensitivity analysis among participants without depression, we still found a detrimental association between prolonged nightly fasting and physical function; therefore, this association was not totally explained by the effect of depression on eating patterns. Whether other causes for the anorexia of ageing may explain prolonged nightly fasting among our study participants and are ultimate responsible of the association found will require additional research.

A variety of intermittent fasting regimens such as time-restricted fasting, periodic fasting, complete alternate-day fasting, modified fasting regimens and religious fasting have been associated with health benefits, including improvements in glucose regulation, blood pressure and heart rate^(2,11,12,16–18)^. There are also evidence of a beneficial effect of these regimes on stress resistance pathways, including increased expression of antioxidant defences, DNA repair, protein quality control, mitochondrial biogenesis and autophagy and down-regulation of inflammation^(10)^. However, intermittent fasting has been mostly studied as a practical approach to restrict energy intake for weight loss^(13)^. Thus, the extrapolation of the health benefits of common intermittent fasting to the general population is difficult. In addition, since prolonged nightly fasting is unintentional, assumption of similar effects of intermittent fasting and prolonged nightly fasting is un-appropriated.

One of the strengths of this study was the assessment of fasting time through a validated diet history that collected habitual diet information in a 24-h format. Another strength was the adjustment for many potential confounders, including sleep duration, energy intake, protein intake, co-morbidities and physical activity. Additionally, the use of an accelerometer was a more reliable way to assess physical activity than self-reported questionnaires, since it provided objective measures on 24-h activity cycle.

On the other hand, the main limitation of this study was the cross-sectional design, which does not allow for causal inference. The possibility for reverse causation in the interpretation of the results is plausible; for example, it is possible that prolonged nightly fasting only reflects a situation in which participants have already developed certain degree of disability that force them to stay longer time in bed, needing to wait for external help to get up and eat. Another limitation of this study is that weight changes associated with fasting could not have been assessed because of the study design; thus, whether weight loss is a mediator in the association between prolonged nightly fasting and ILEF is uncertain. Moreover, as in all population studies, some residual and unmeasured confounding cannot be ruled out, despite the associations between the main variables studied were adjusted for an important number of potential confounders. Last, since the cross-sectional design in this study, OR obtained should not be interpreted as proxies of relative risk for incidence of ILEF.

In conclusion, fasting time ≥12 h/d was associated with a higher frequency of ILEF, balance impairment and difficulty to rise from a chair in older adults, independent of physical activity level. These results suggest a detrimental effect of prolonged nightly fasting in older adults. Further longitudinal studies of fasting in relation to ILEF would be valuable to understand if this is a causal association.

## Supplementary material

For supplementary material accompanying this paper visit http://dx.doi.org/10.1017/S0007114520005218. click here to view supplementary material

## References

[1] Barnosky AR , Hoddy KK , Unterman TG , et al. (2014) Intermittent fasting vs daily calorie restriction for type 2 diabetes prevention: a review of human findings. Transl Res 164, 302–311. 24993615 10.1016/j.trsl.2014.05.013

[2] Mattson MP , Allison DB , Fontana L , et al. (2014) Meal frequency and timing in health and disease. Proc Natl Acad Sci U S A 111, 16647–16653. 25404320 10.1073/pnas.1413965111 PMC4250148

[3] Tinsley GM & La Bounty PM (2015) Effects of intermittent fasting on body composition and clinical health markers in humans. Nutr Rev 73, 661–674. 26374764 10.1093/nutrit/nuv041

[4] Melkani GC & Panda S (2017) Time-restricted feeding for prevention and treatment of cardiometabolic disorders. J Physiol 595, 3691–3700. 28295377 10.1113/JP273094 PMC5471414

[5] Ahmet I , Wan R , Matsson MP , et al. (2005) Cardioprotection by intermittent fasting in rats. Circulation 112, 3115–3121. 16275865 10.1161/CIRCULATIONAHA.105.563817

[6] Goodrick CL , Ingram DK , Reynolds MA , et al. (1990) Effects of intermittent feeding upon body weight and lifespan in inbred mice: interaction of genotype and age. Mech Ageing Dev 55, 69–87. 2402168 10.1016/0047-6374(90)90107-q

[7] Marianna P , Iolanda C , Andrea E , et al. (2019) Effects of time-restricted feeding on body weight and metabolism. A systematic review and meta-analysis. Rev Endocr Metab Disord 21, 17–33. 10.1007/s11154-019-09524-w 31808043

[8] Moro T , Tinsley G , Bianco A , et al. (2016) Effects of eight weeks of time-restricted feeding (16/8) on basal metabolism, maximal strength, body composition, inflammation, and cardiovascular risk factors in resistance-trained males. J Transl Med 14, 290. 27737674 10.1186/s12967-016-1044-0 PMC5064803

[9] Hutchinson AT , Regmi P , Manoogian ENC , et al. (2019) Time-restricted feeding improves glucose tolerance in men at risk for type 2 diabetes: a randomized crossover trial. Obesity 27, 724–732. 31002478 10.1002/oby.22449

[10] De Cabo R & Mattson MP (2019) Effects of intermittent fasting on health, aging, and disease. N Engl J Med 381, 2541–2551. 31881139 10.1056/NEJMra1905136

[11] Anton SD , Moehl K , Donahoo WT , et al. (2018) Flipping the metabolic switch: understanding and applying the health benefits of fasting. Obesity 26, 254–268. 29086496 10.1002/oby.22065 PMC5783752

[12] Di Francesco A , Di Germanio C , Bernier M , et al. (2018) A time to fast. Science 362, 770–775. 30442801 10.1126/science.aau2095 PMC8504313

[13] Anton SD , Lee SA , Donahoo WT , et al. (2019) The effects of time restricted feeding on overweight, older adults: a pilot study. Nutrients 11, 1500. 10.3390/nu11071500 PMC6682944 31262054

[14] Tinsley GM , Forsse JS , Butler NK , et al. (2017) Time-restricted feeding in young men performing resistance training: a randomized controlled trial. Eur J Sport Sci 17, 200–207. 27550719 10.1080/17461391.2016.1223173

[15] Tinsley GM , Moore ML , Graybeal AJ , et al. (2019) Time-restricted feeding plus resistance in active females: a randomized trial. Am J Clin Nutr 110, 628–640. 31268131 10.1093/ajcn/nqz126 PMC6735806

[16] Antunes F , Erustes AG , Costa AJ , et al. (2018) Autophagy and intermittent fasting: the connection for cancer therapy? Clinics (Sao Paulo) 73, e814s. 30540126 10.6061/clinics/2018/e814s PMC6257056

[17] Patterson RE & Sears DD (2017) Metabolic effects of intermittent fasting. Annu Rev Nutr 37, 371–393. 28715993 10.1146/annurev-nutr-071816-064634 PMC13170603

[18] Froy O & Miskin R (2010) Effect of feeding regimens on circadian rhythms: implications for aging and longevity. Aging 2, 7–27. 20228939 10.18632/aging.100116 PMC2837202

[19] Cruz-Jentoft AJ & Sayer AA (2019) Sarcopenia. Lancet 393, 2636–2646. 31171417 10.1016/S0140-6736(19)31138-9

[20] Rodríguez-Artalejo F , Graciani A , Guallar-Castillón P , et al. (2011) Rationale and methods of the Study on Nutrition and Cardiovascular Risk in Spain (ENRICA). Rev Española Cardiol 64, 876–882. 10.1016/j.recesp.2011.05.019 21821340

[21] Cabanas-Sánchez V , Esteban-Cornejo I , Migueles JH , et al. (2019) Twenty four-hour activity cycle in older adults using wrist-worn accelerometers: the seniors-ENRICA-2 study. Scand J Med Sci Sports 30, 700–708. 10.1111/sms.13612 31834945

[22] Guallar-Castillón P , Sagardui-Villamor J , Balboa-Castillo T , et al. (2014) Validity and reproducibility of a Spanish dietary history. PLOS ONE 9, e86074. 24465878 10.1371/journal.pone.0086074 PMC3896441

[23] Moreiras O , Carbajal A , Cabrera L , et al. (2007) Tablas de composición de alimentos. 11ª Edición (*Food Composition Table*, 11th ed.). Madrid: Pirámide.

[24] Schröder H , Fitó M , Estruch R , et al. (2011) A short screener is valid for assessing Mediterranean diet adherence among older Spanish men and women. J Nutr 141, 1140–1145. 21508208 10.3945/jn.110.135566

[25] Yuan C , Spiegelman D , Rimm EB , et al. (2017) Validity of a dietary questionnaire assessed by comparison with multiple weighed dietary records or 24-hour recalls. Am J Epidemiol 185, 570–584. 28338828 10.1093/aje/kww104 PMC5859994

[26] Guralnik JM , Ferrucci L , Pieper CF , et al. (2000) Lower extremity function and subsequent disability: consistency across studies, predictive models, and value of gait speed alone compared with the short physical performance battery. J Gerontol A Biol Sci Med Sci 55, M221–M231. 10811152 10.1093/gerona/55.4.m221 PMC12149745

[27] Guralnik JM , Ferrucci L , Simonsick EM , et al. (1995) Lower-extremity function in persons over the age of 70 years as a predictor of subsequent disability. N Engl J Med 332, 556–561. 7838189 10.1056/NEJM199503023320902 PMC9828188

[28] Laatar R , Borji R , Baccouch R , et al. (2016) Effects of Ramadan fasting on postural balance and attentional capacities in elderly people. J Nutr Health Aging 20, 553–560.2710279510.1007/s12603-015-0620-y

[29] Laatar R , Baccouch R , Borji R , et al. (2019) Ramadan fasting effects on postural control in the elderly: a comparison between fallers and non-fallers. J Relig Health 58, 28–40. 27804006 10.1007/s10943-016-0323-7

[30] Martens CR , Rossman MJ , Mazzo MR , et al. (2020) Short-term time-restricted feeding is safe and feasible in non-obese healthy midlife and older adults. Geroscience 42, 667–686. 31975053 10.1007/s11357-020-00156-6 PMC7206473

[31] Simonsick EM , Guralnik JM , Volpato S , et al. (2005) Just get out the door! The importance of walking outside the home for maintaining mobility: findings from the Women’s Health and Aging Study. J Am Geriatr Soc 53, 198–203. 15673341 10.1111/j.1532-5415.2005.53103.x

[32] Daly RM , Ahlborg HG , Ringsberg K , et al. (2008) Association between changes in habitual physical activity and changes in bone density, muscle strength, and functional performance in elderly men and women. J Am Geriatr Soc 56, 2252–2260. 19016934 10.1111/j.1532-5415.2008.02039.x

[33] Yorston LC , Kolt GS & Rosenkranz RR (2012) Physical activity and physical function in older adults: the 45 and up study. J Am Geriatr Soc 60, 719–725. 22486736 10.1111/j.1532-5415.2012.03906.x

[34] Coelho-Júnior HJ , Milano-Teixeira L , Rodrigues B , et al. (2018) Relative protein intake and physical function in older adults: a systematic review and meta-analysis of observational studies. Nutrients 10, 1330. 10.3390/nu10091330 PMC6163569 30235845

[35] Hruby A , Sahni S , Bolster D , et al. (2020) Protein intake and functional integrity in aging: the Framingham Heart Study offspring. J Gerontol A Biol Sci Med Sci 75, 123–130. 30247514 10.1093/gerona/gly201 PMC6909900

[36] Bauer J , Biolo G , Cederholm T , et al. (2013) Evidence-based recommendations for optimal dietary protein intake in older people: a position paper from the PROT-AGE study group. J Am Med Dir Assoc 14, 542–559. 23867520 10.1016/j.jamda.2013.05.021

[37] Scott D , Blizzard L , Fell J , et al. (2010) Associations between dietary nutrient intake and muscle mass and strength in community-dwelling older adults: the Tasmanian Older Adult cohort study. J Am Geriatr Soc 58, 2129–2134. 21054294 10.1111/j.1532-5415.2010.03147.x

[38] Rizzoli R , Biver E , Bonjour JP , et al. (2018) Benefits and safety of dietary protein for bone health—an expert consensus paper endorsed by the European Society for Clinical and Economical Aspects of Osteopororosis, Osteoarthritis, and Musculoskeletal Diseases and by the International Osteoporosis Foundation. Osteoporos Int 29, 1933–1948. 29740667 10.1007/s00198-018-4534-5

[39] Otsuka R , Tange C , Tomida M , et al. (2019) Dietary factors associated with the development of physical frailty in community-dwelling older adults. J Nutr Health Aging 23, 89–95.3056907510.1007/s12603-018-1124-3

[40] Bradlee ML , Mustafa J , Singer MR , et al. (2017) High-protein foods and physical activity protect against age-related muscle loss and functional decline. J Gerontol A Biol Sci Med Sci 73, 88–94. 28549098 10.1093/gerona/glx070 PMC5861873

[41] Institute of Medicine (2005) Dietary Reference Intakes for Energy, Carbohydrate, Fiber, Fat, Fatty Acids, Cholesterol, Protein, and Amino Acids (Macronutrients). Washington, DC: The National Academies Press.

[42] Estrada-DeLeón DB , Struijk EA , Caballero F , et al. (2020) Distribution of protein intake across meals and lower-extremity functioning in community-dwelling Spanish older adults: a prospective cohort study. Eur J Nutr (epublication ahead of print version 16 May 2020). 10.1007/s00394-020-02273-6 32417947

[43] Martone AM , Onder G , Vetrano DL , et al. (2013) Anorexia of aging: a modifiable risk factor for frailty. Nutrients 5, 4126–4133. 24128975 10.3390/nu5104126 PMC3820063

[44] Landi F , Calvani R , Tosato M , et al. (2016) Anorexia of aging: risk factors, consequences, and potential treatments. Nutrients 8, 69. 26828516 10.3390/nu8020069 PMC4772033

[45] İlhan B , Bahat G , Erdoğan T , et al. (2019) Anorexia is independently associated with decreased muscle mass and strength in community dwelling older adults. J Nutr Health Aging 23, 202–206.3069763110.1007/s12603-018-1119-0

