# Supplementary material for: Prolonged nightly fasting and lower-extremity functioning in community-dwelling older adults
Source: Br J Nutr. 2020 Dec 29;126(9):1347–54. doi: 10.1017/S0007114520005218 (PMC8505711; doi:10.1017/S0007114520005218)
Supplement: Supplementary file 1 [file S0007114520005218sup.zip › S0007114520005218sup001.docx]

Exclusions

No information on timing of food consumption (n=1,541).

Implausible energy intake (n=1).

No information on educational level (n=1).

No performance of the Short Physical Performance Battery (n=454).

No information on accelerometry (n=50).

Analytical sample

1,226 participants

2016-2017 Seniors-ENRICA II cohort

3,273 participants ≥64 years

**Seniors-ENRICA II study**

**Supplemental Figure 1.** Flow-chart
