# Supplementary material for: Prolonged nightly fasting and lower-extremity functioning in community-dwelling older adults
Source: Br J Nutr. 2020 Dec 29;126(9):1347–54. doi: 10.1017/S0007114520005218 (PMC8505711; doi:10.1017/S0007114520005218)
Supplement: Supplementary file 1 [file S0007114520005218sup.zip › S0007114520005218sup002.docx]

Supplemental Table 1. Odds ratios (95% confidence interval) for the association between fasting time categories and impaired lower-extremity function (ILEF), balance impairment, difficulty to rise from a chair, and slow gait among participants without depression (N=1,130).

|  | | ≤9h fasting | 10-11h fasting | ≥12 h fasting | p trend |
| --- | --- | --- | --- | --- | --- |
|  | |  |  |  |  |
| n | | 541 | 364 | 225 |  |
| ILEF, n cases | | 64 | 77 | 64 |  |
|  | Model 1 | 1.00 | 2.14 (1.47-3.11) | 2.65 (1.77-3.97) | <0.001 |
|  | Model 2 | 1.00 | 2.25 (1.52-3.32) | 2.81 (1.83-4.30) | <0.001 |
|  | Model 3 | 1.00 | 2.28 (1.54-3.39) | 2.73 (1.77-4.19) | <0.001 |
| Balance impairment, n cases | | 37 | 29 | 39 |  |
|  | Model 1 | 1.00 | 1.27 (0.76-2.14) | 2.59 (1.57-4.27) | <0.001 |
|  | Model 2 | 1.00 | 1.24 (0.72-2.13) | 2.68 (1.59-4.53) | <0.001 |
|  | Model 3 | 1.00 | 1.22 (0.71-2.10) | 2.56 (1.52-4.33) | 0.001 |
| Difficulty to raise from  a chair, n cases | | 345 | 243 | 154 |  |
|  | Model 1 | 1.00 | 1.18 (0.89-1.56) | 1.24 (0.88-1.73) | 0.16 |
|  | Model 2 | 1.00 | 1.26 (0.94-1.69) | 1.29 (0.91-1.83) | 0.09 |
|  | Model 3 | 1.00 | 1.29 (0.96-1.73) | 1.29 (0.91-1.83) | 0.09 |
| Slow gait, n cases | | 135 | 87 | 75 |  |
|  | Model 1 | 1.00 | 0.92 (0.67-1.26) | 1.26 (0.89-1.79) | 0.31 |
|  | Model 2 | 1.00 | 0.89 (0.65-1.24) | 1.28 (0.89-1.84) | 0.29 |
|  | Model 3 | 1.00 | 0.90 (0.65-1.24) | 1.27 (0.89-1.83) | 0.31 |

Model 1: OR (95% CI) adjusted for sex, age and energy intake (quintiles of kcal/day).

Model 2: OR (95% CI) additionally adjusted for educational level (≤primary, secondary, or university), smoking status (never, former, current smoker), sedentary behavior (tertiles of h/week watching TV), alcohol intake (quintiles of g/day), BMI (<25, 25-29.9, ≥30 kg/m²), morbidity (musculoskeletal disease, cardiovascular disease, cancer, diabetes, and chronic lung disease), sleep duration (tertiles of hours/d), protein intake (quintiles of g/d), and for MEDAS score (tertiles).

Model 3: OR (95% CI) additionally adjusted for physical activity (tertiles of METs-h/week).

These analyses have been performed without those participants with depression.
